# Unveiling the Spectrum of Otorhinolaryngological Manifestations in Siblings With Bardet-Biedl Syndrome: A Report of Two Cases

**DOI:** 10.7759/cureus.66233

**Published:** 2024-08-05

**Authors:** Karthikeyan Padmanabhan, Sharad Ashish, Neelima Vijayan, Swaati Mukilan Hemanthkumar

**Affiliations:** 1 Otolaryngology - Head and Neck Surgery, Mahatma Gandhi Medical College and Research Institute, Pondicherry, IND; 2 Otolaryngology - Head and Neck Surgery, Srinivasan Medical College and Hospital, Tiruchirappalli, IND

**Keywords:** bardet–biedl syndrome, adenoid regrowth, adult tonsils, difficult airway intubation, paediatric otorhinolaryngology

## Abstract

Bardet-Biedl syndrome (BBS) is a rare autosomal recessive ciliopathy characterized by diverse multisystem manifestations. This report discusses the unique otorhinolaryngological challenges faced by two pediatric siblings diagnosed with BBS. Case 1 involves a child with a history of chronic snoring, delayed developmental milestones, and a low intelligence quotient (IQ). The patient presented with obesity, retinitis pigmentosa, and a rare bifid epiglottis, adding to the complexity. Adenotonsillectomy was indicated due to chronic adenotonsillitis, but the presence of a grade 4 Mallampati score and restricted mouth opening required meticulous planning by the surgical and anesthesia teams. The collaborative approach led to a successful procedure, emphasizing the importance of interdisciplinary coordination in managing complex cases. Case 2, the younger sibling, presented with disturbed sleep cycles, mouth breathing, and difficulty swallowing. Adenotonsillectomy was performed for chronic adenotonsillitis, providing relief initially. However, recurrent adenoid hypertrophy, covering 90% of choanae, manifested two years later. The case highlights the need for long-term follow-up and raises questions about the underlying mechanisms contributing to recurrent adenoid hypertrophy in BBS. These cases underscore the rarity and intricacy of otorhinolaryngological manifestations in BBS, emphasizing the importance of comprehensive and multidisciplinary management. The challenges posed by anatomical abnormalities and recurrent adenoid hypertrophy necessitate ongoing research for effective long-term strategies in treating these complex genetic conditions. These findings contribute to the limited literature on BBS within the otorhinolaryngology domain and underscore the significance of continued collaboration and research efforts in optimizing patient care.

## Introduction

Bardet-Biedl syndrome (BBS) is an autosomal recessive multisystem non-motile ciliopathy primarily marked by retinal cone-rod dystrophy, hypogonadotropic hypogonadism, renal malformations and/or renal parenchymal disease, and/or genitourinary malformations, postaxial polydactyly, obesity, and related complications. The structure and function of the motile cilia are largely normal in BBS, but patients who have it have a higher probability of having symptoms of the motile ciliopathies, such as thoracoabdominal laterality abnormalities, asthma, otitis media, and newborn respiratory distress [1]. The clinical spectrum of BBS patients, however, is broad and can also include hearing loss, delayed speech, dental abnormalities, a high-arched palate, heart anomalies, and diabetes mellitus, among other medical conditions [2]. According to Forsythe et al. [3], the presence of either four main characteristics or three major features and two minor features can be used for establishing a clinical diagnosis of BBS [3]. In the otorhinolaryngology literature, case reports pertaining to BBS are very limited, and not many cases have been reported from the Indian subcontinent. Here, we report the cases of two siblings who both suffer from BBS and had presented to ENT outpatient department with otorhinolaryngological manifestations, and the course of action of their management has been discussed here.

## Case presentation

Case 1

**Figure 1:**
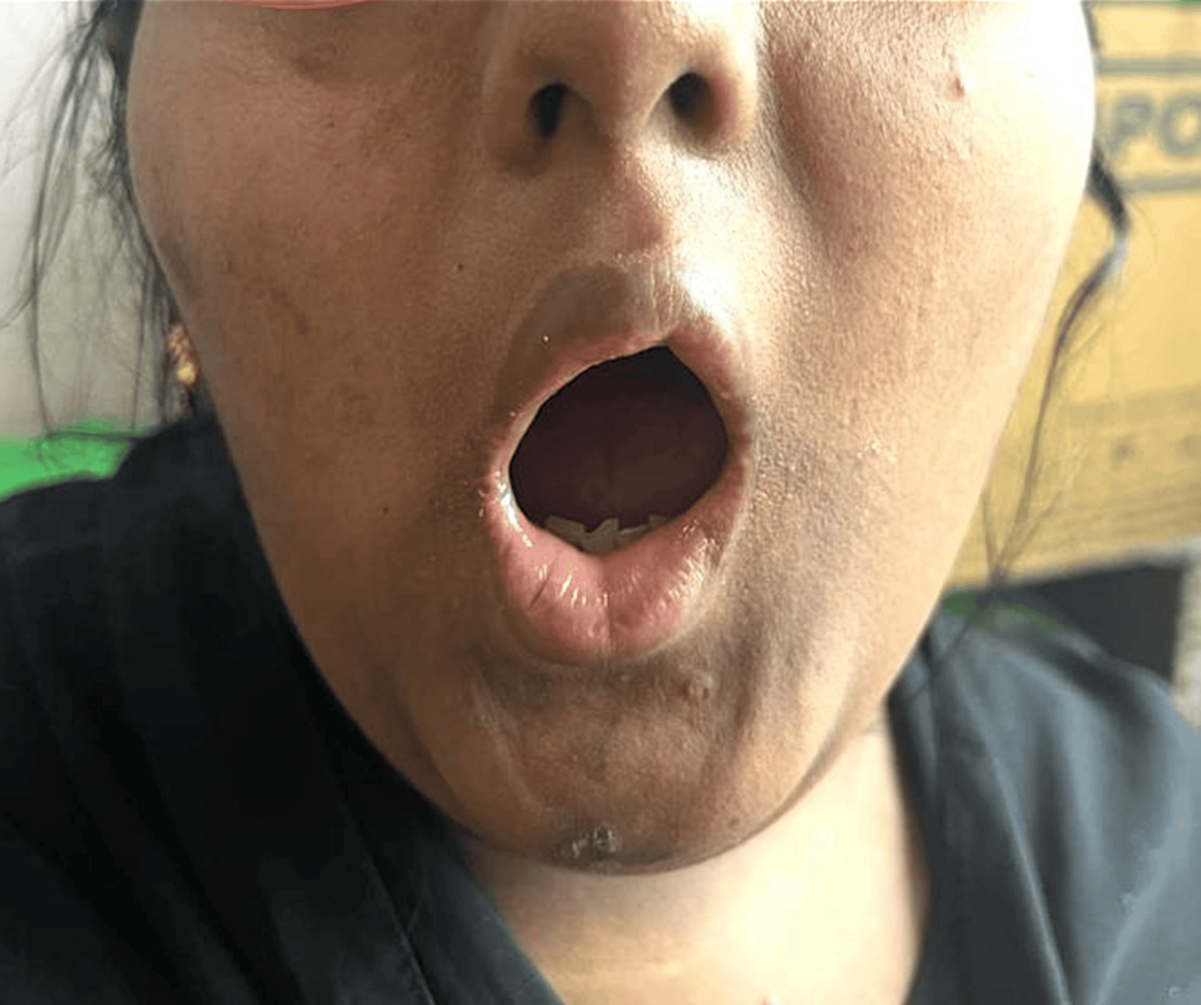
Restricted mouth opening in Case 1 (grade 4 Mallampati score)

**Figure 2:**
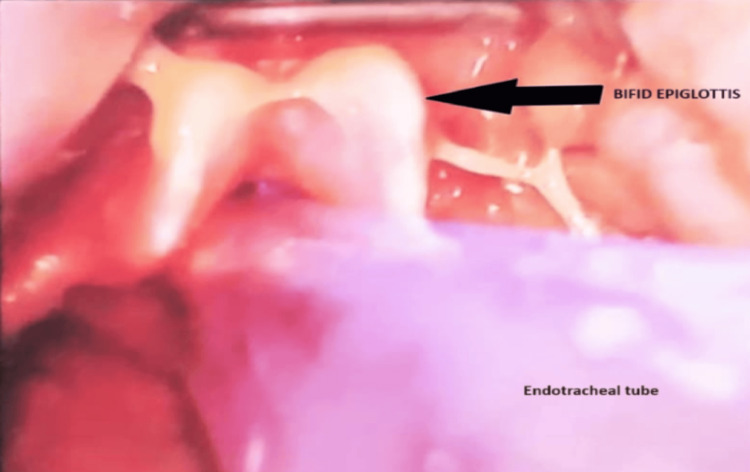
Bifid epiglottis (marked by an arrow)

**Figure 3:**
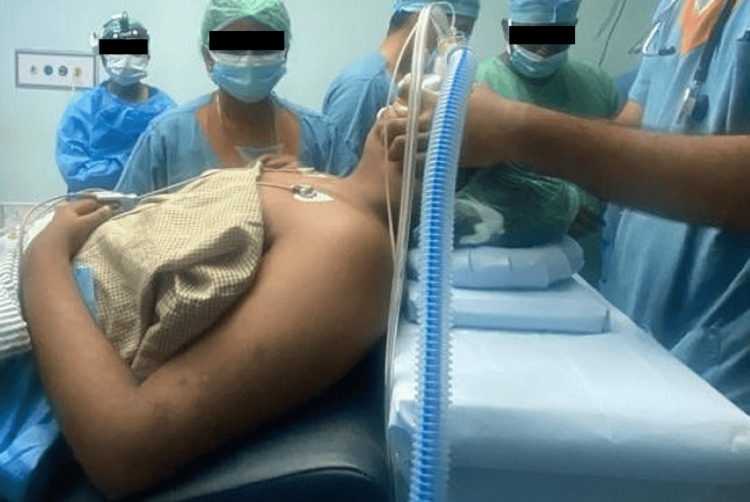
Position used while giving anesthesia to the patient (Case 1)

The patient, a 17-year-old female, the first child of non-consanguineous parents, arrived at the ENT outpatient department with a history of chronic snoring and mouth breathing persisting for eight years. Furthermore, the child had experienced delayed developmental milestones compared to peers, with a low intelligence quotient (IQ) of 42, indicating mild-to-moderate intellectual disability. Night blindness had been present since the age of two, while the general examination revealed obesity with a BMI of 33, a blood pressure reading of 124/72 mmHg, and a pulse rate of 82 beats per minute. Fundus examination revealed retinitis pigmentosa, a degenerative eye disorder. Local examination of the oral cavity and throat unveiled restricted mouth opening (Figure 1), with crowding of teeth and grade 3 tonsillar enlargement, with 100% adenoid obstruction of the choanae, as confirmed by diagnostic nasal endoscopy. An abdominal ultrasound scan indicated the presence of an infantile uterus, while endocrinological evaluation revealed hypogonadotropic hypogonadism. Video laryngoscopic examination incidentally discovered a bifid epiglottis (Figure 2), adding further rarity to this already unique case. Hearing evaluations, including the brainstem evoked response audiometry (BERA) assessment, showed normal hearing, while impedance assessment revealed a C-type curve on the left side. The patient's diagnosis of BBS, accompanied by chronic adenotonsillitis, necessitated surgical intervention in the form of adenotonsillectomy. However, the grade 4 Mallampati score and restricted mouth opening posed significant challenges for the surgical and anesthesia teams. Operating in such conditions required meticulous planning and careful execution to ensure the patient's safety and a successful outcome. The surgical and anesthesia teams employed a collaborative approach to address the challenges presented by the patient's condition. Preoperative evaluations were conducted to assess the patient's overall health status and to identify potential risks associated with the surgery. Alternative strategies for airway management were prepared in case of difficulties during intubation. During the surgery, the patient's positioning was carefully managed to optimize airway alignment (Figure 3). Continuous monitoring of the patient's vital signs and close communication between the surgical and anesthesia teams ensured a smooth surgical procedure. Postoperatively, the patient remained stable, exhibiting a successful recovery.

Case 2

A 10-year-old male patient, diagnosed with BBS based on the Forsythe and Beales criteria [3], exhibited central obesity, cognitive impairment, chronic kidney disease, metabolic syndrome, and behavioral abnormalities. He was the second child of a non-consanguineous marriage and the younger brother of the first patient. He presented to the ENT outpatient department with complaints of disturbed sleep cycles, mouth breathing, and difficulty swallowing for the past two years. The patient had a history of frequent upper respiratory tract infections (URTIs).

**Figure 4:**
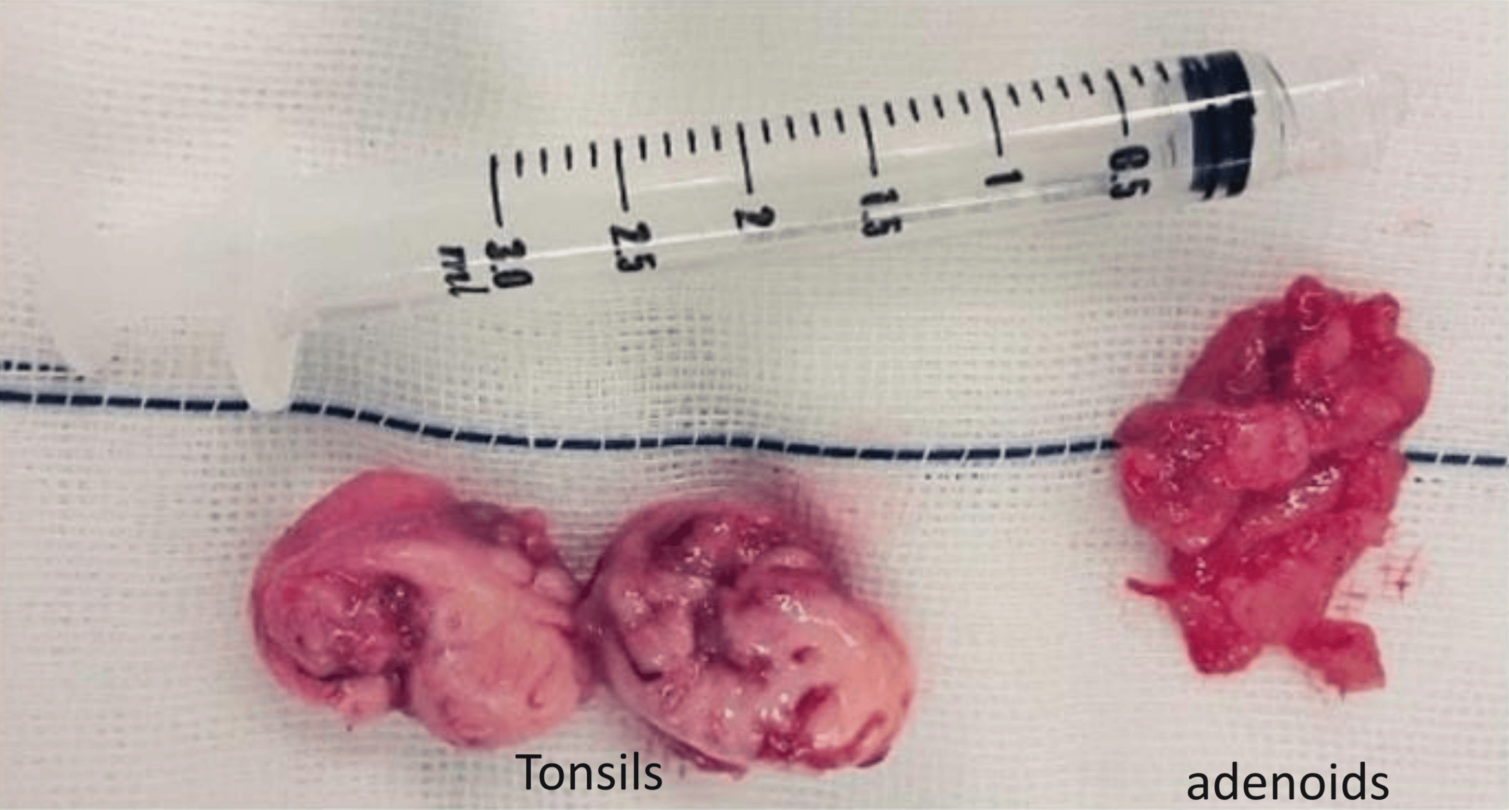
Excised specimen of a pair of tonsils (left) and adenoids (right)

On general examination, the patient had a BMI of 32.7, indicating obesity, and his blood pressure was within normal limits (110/70 mmHg). Local examination of the oral cavity and throat revealed grade 3 tonsillar enlargement. Diagnostic nasal endoscopy showed grade III adenoid hypertrophy. The patient was diagnosed with chronic adenotonsillitis and scheduled for adenotonsillectomy. The surgery was performed without complications, and the postoperative period was uneventful (Figure 4). The patient experienced relief from symptoms and improved sleep patterns following the procedure.

**Figure 5:**
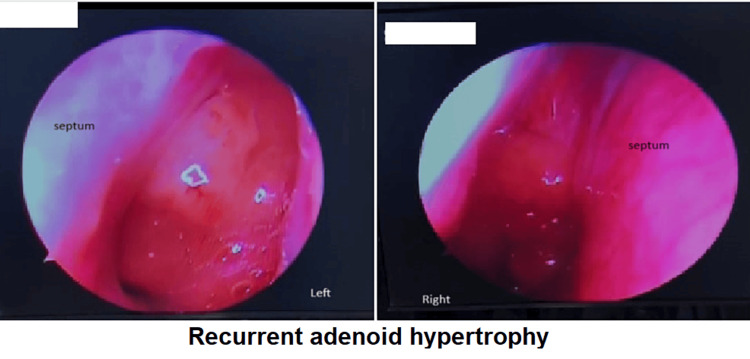
Recurrent growth of adenoid tissue

Two years later, the patient returned with complaints of mouth breathing for the past month. Repeat diagnostic nasal endoscopy revealed adenoid enlargement, covering 90% of both choanae (Figure 5).

## Discussion

As a multisystem non-motile ciliopathy, BBS is primarily distinguished by renal malformations and/or renal parenchymal disease, hypogonadotropic hypogonadism and/or genitourinary malformations, postaxial polydactyly, obesity and its complications, and retinal cone-rod dystrophy. Other eye abnormalities (strabismus, astigmatism, cataracts), subtle craniofacial dysmorphisms (crowding, hypodontia, high-arched palate), hearing loss, anosmia, musculoskeletal abnormalities, dermatologic abnormalities, and neurodevelopmental abnormalities (mild hypertonia, ataxia/poor coordination/imbalance, developmental delay(s), seizure) can also be present in people with BBS [1,4]. Globally, approximately 70-80% of BBS cases can be attributed to mutations in genes ranging from BBS1 to BBS18. However, in the Indian population, the prevalence of BBS1 and BBS10 mutations in the diagnosis is merely 7% and 10%, respectively. On the other hand, BBS3 (14%), BBS9 (10%), and BBS6 (10%) mutations are more commonly observed [5]. Due to the rarity of BBS, there is a dearth of evidence-based recommendations for treating these individuals, particularly from the standpoint of the otolaryngologist [2]. Among the limited literature available from the Indian subcontinent, Singh et al. presented a case report discussing the occurrence of otorhinological manifestations, such as sensorineural hearing loss and chronic tonsillitis, in patients diagnosed with BBS [6]. In our particular case, the presence of a Mallampati score of 4 presented challenges for both the anesthetist and surgeons in ensuring a successful surgical outcome of adenotonsillectomy. However, despite the difficulty, the surgery was ultimately accomplished. Additionally, the rarity of our case is further emphasized by the fact that the occurrence of a bifid epiglottis in BBS has been reported in only nine patients between 1990 and 2022 [7]. Cronjé, in a separate report, has documented the presence of a bifid epiglottis in BBS patients who did not exhibit any symptoms, mirroring the observation made in our own case [8]. The occurrence of recurrent adenoid hypertrophy is not a recognized complication in individuals with BBS, and the underlying causes and mechanisms responsible for adenoid hypertrophy in BBS patients remain uncertain. The high prevalence of obesity in BBS patients may contribute to the development of adenoid hypertrophy, as adipose tissue can release proinflammatory cytokines [9]. In our case, despite the initial adenotonsillectomy, the patient developed recurrent adenoid hypertrophy, indicating the need for vigilant long-term follow-up. Repeat adenoidectomy may be necessary in cases of recurrent adenoid hypertrophy to alleviate symptoms and improve the patient's quality of life.

## Conclusions

In conclusion, these two case reports shed light on the rare and intricate medical challenges associated with BBS in pediatric patients. In the first case, a child diagnosed with BBS, accompanied by chronic adenotonsillitis, presented a multitude of symptoms requiring an adenotonsillectomy. Despite the complexities posed by the patient's grade 4 Mallampati score, successful surgery and a stable postoperative period were achieved through collaborative efforts. This underscores the significance of interdisciplinary coordination and specialized expertise in managing complex cases to optimize patient outcomes. The second case report emphasizes the difficulties encountered in managing recurrent adenoid hypertrophy in pediatric patients with BBS. Long-term follow-up and regular evaluation of the upper respiratory tract are crucial for promptly identifying and addressing episodes of recurrent adenoid hypertrophy. Further research is necessary to gain a deeper understanding of the underlying mechanisms and develop effective management strategies for this recurring condition in individuals with BBS. These cases highlight the importance of a comprehensive and multidisciplinary approach to the management of BBS, considering the diverse range of symptoms and potential complications involved. Continued collaboration between healthcare professionals, along with long-term monitoring and research efforts, are vital in enhancing the care and outcomes for patients with this complex genetic disorder.
